# Life cycle approach as a tool for assessing municipal biowaste treatment units: A systematic review

**DOI:** 10.1177/0734242X251326866

**Published:** 2025-03-27

**Authors:** Laís Fabiana Serafini, Paulo José Gomes Monteiro Praça, Fernando González-Andrés, Artur Gonçalves

**Affiliations:** 1 CIMO, LA SusTEC, Instituto Politécnico de Bragança, Campus de Santa Apolónia, 5300-253 Bragança, Portugal; 2 Escola Superior de Comunicação, Administração e Turismo do Instituto Politécnico de Bragança, Bragança, Portugal; 3 Chemical, Environmental and Bioprocess Engineering Group, Institute of Research and Innovation in Engineering (I4), University of León, Av. Portugal, 41, 24009, León, Spain

**Keywords:** Life cycle assessment, composting, anaerobic digestion, global warming potential, waste management, impact categories

## Abstract

Biowaste is an increasingly relevant environmental issue worldwide, causing significant environmental, economic and social impacts. Effective strategies are crucial to mitigate impacts, maximising biowaste’s valorisation. This article presents a systematic literature review on using life cycle assessment (LCA) to evaluate municipal biowaste treatment facilities. The primary objective was to analyse how LCA is applied to assess the environmental efficiency of mechanical and biological treatment involving composting and anaerobic digestion (AD)-based systems. The article addressed the methodological heterogeneity across previous LCA studies, identifying critical gaps and challenges regarding standardisation and result comparability. It underscores the importance of accurately considering environmental indicators and emission factors, as these significantly affect overall LCA outcomes. Results show that most publications focus on Europe and Asia, highlighting a research gap in regions like Africa. The organic fraction municipal solid waste is the predominant feedstock, and 1 tonne of biowaste was the frequently used functional unit, reflecting the upstream impacts of waste. The most recurrent system boundary was the cradle-to-grave, offering a comprehensive analysis as it covers all stages of biowaste treatment from collection to disposal. The studies highlight the environmental benefits of AD-based systems through energy production compensations, particularly in reducing global warming potential, compared with other treatment operations such as landfills. While replacing mineral fertilisers with digestate and compost is very well discussed, it raises concerns about heavy metal content and nutrient availability. Therefore, selective collection of organic waste is crucial to improve compost quality and AD efficiency, though it increases transportation costs.

## Introduction

The growing increase in biowaste generation is a multifactorial issue mainly due to growing urbanisation and the changes in the population’s consumption profile (Omenesa Idris et al., 2022). With population growth, the demand for food and consumption increases, accelerating industrial processing and agricultural activities (Gaur et al., 2020). Globally, approximately one-third of all food produced for human consumption is lost or wasted, with a significant portion of this loss occurring before it even reaches consumers (FAO, 2019). About 14% of food is lost between the post-harvest and retail stages (FAO, 2019). In the EU-28 alone, approximately 86 million tonnes of biological waste are produced annually (EEA, 2020).

Biowaste is defined as biodegradable waste, including food waste from households, restaurants, offices, food processing plants and waste from gardens and parks maintenance (EEA, 2020). Selective collection planning and adequate treatment technology are fundamental to preventing increased environmental impacts from biowaste management (Cheng et al., 2022; Mayanti and Helo, 2023). However, this particular waste is often complex as it has diverse chemical, physical and biodegradation characteristics, and its management options may generate high amounts of ammonia emissions, greenhouse gases (GHG) and the release of heavy metals, leading to air, water and soil contamination (Simcock et al., 2019).

The two most conventional solutions for biowastes are landfills and incineration, options that raise environmental, health and social concerns due to GHG emissions, leachate production, abiotic depletion, land occupation and lack of economic sustainability (Fernández-Domínguez et al., 2020; Medeiros et al., 2022). Other more valuable techniques offer biological transformations, such as mechanical biological treatment (MBT) followed by composting and anaerobic digestion (AD), both reducing the impacts associated with biowaste management and adding value by generating by-products (compost and/or energy) and promoting the circular bioeconomy (Sherwood, 2020; Weidner et al., 2020). Municipal waste management systems include collecting, sorting and processing different types of solid waste. These systems also include landfills, recycling centres, waste-to-energy plants and biological treatment operations, entailing many environmental and economic impacts that must be adequately addressed (Laso et al., 2019; Saleh and Hassan, 2021).

The compost produced through the biological treatment of biowaste has the potential to be an agronomic value product because it is rich in nutrients and organic matter, capable of improving soil structure, enhancing carbon storage capacity in the soil, climate change mitigation and reducing the use of synthetic fertilisers (Lejarazu-Larrañaga et al., 2020; Pergola et al., 2020). The production of synthetic fertilisers from nitrogen and phosphate requires significant energy from non-renewable sources that increase the global warming potential (GWP), which could be reduced using compost (Arrobas et al., 2022; Sherwood, 2020). Furthermore, the growing use of synthetic fertilisers over the past decades has caused substantial damage to soils’ health and adverse effects that include land degradation, eutrophication and contribution to global warming (GHG emissions) that can be bioremediated with repeated or long-term application of organic composts (Arrobas and Ângelo Rodrigues, 2013; Serafini et al., 2024; Sharma et al., 2019; Urbano et al., 2022).

The life cycle assessment (LCA) approach has been used to estimate the environmental impacts caused by biowaste and their treatment techniques, which can be followed by life cycle costing (LCC) to measure economic performance indicators (Paes et al., 2020; Tomić and Schneider, 2020). The main impact categories used to assess biowaste and its treatment techniques are GWP, acidification potential (AP), human toxicity potential (HTP), eutrophication potential (EP) and ozone layer depletion (OLD) (Iqbal et al., 2020; Zhang et al., 2021). The leaching nitrate and phosphate contribute directly to the EP impact category, as the emission of ammonia is the main contributor to AP, and the GHGs (methane, nitrous oxide and carbon dioxide) are contributors to GWP (Serafini et al., 2023). The main aspects that contribute to the lack of economic sustainability of the biowaste management analysed by the LCC are generally the high volume of biowaste deposited in landfills, without alternative treatments for non-recyclable materials, the low rate of its selective collection and the low performance of biological treatments (Fernández-Braña et al., 2020).

When studied from an LCA perspective, biowaste should be regarded as a resource, as reuse and recycling are vital to decreasing the input and output fluxes into the economy (Geng et al., 2019; Ripa et al., 2017). Therefore, adequately managing biowaste in an environmentally and economically appropriate approach is extremely important to mitigate environmental pollution, guarantee ecological living standards and promote a circular economy (Saravanan et al., 2023; Velenturf et al., 2019).

Therefore, we comprehensively scrutinise the life cycle methodologies employed in municipal biowaste in investigation research. The primary objective was to discern the relevant factors and processes determining the environmental efficiency of composting and AD within these contexts. Subsequently, we analyse how these studies have delineated the boundaries of municipal biowaste systems, sourced data and determined the main impact assessment methods and categories, providing insights into the main results, primarily GWP results and key findings.

## Materials and methods

This systematic review was initiated by carefully choosing the best combination of search terms to refine the search and get the most accurate results. The filters used for this search consisted of selecting articles published from 2018 to 2023 to ensure the updated studies yielded more information aligned with the defined scope for the review and only included original articles in English. The search terms were entered in the Scopus and Web of Science (WoS) databases in the search within article titles, abstracts and keywords:

- ‘Life cycle*’ OR ‘LCA’ OR ‘LCC’
- (‘mechanic* biologic* treatment’ OR ‘MBT’) OR (composting AND ‘anaerobic digestion’)
- ‘municipal organic wast*’ OR ‘municipal bio*wast*’ OR ‘Urban biodegradable wast*’ OR ‘residential wast*’ OR ‘organic* fraction municipal* solid wast*’ OR ‘OFMSW’ OR ‘organic fraction’

Following this search, 25 articles were found in the Scopus and 35 in the WoS. Afterwards, the search results were screened using the Rstudio tool and the Bibliometrix package (Aria and Cuccurullo, 2017; Pagani et al., 2015). PRISMA statement was used to conduct this systematic review and apply the defined exclusion criteria for filtering the most relevant studies (Supplemental Figure S1) (Page et al., 2021).

There were 17 duplicate documents removed, and the rest, 43, were inserted in the StArt tool (Fabbri et al., 2016) for selection, applying exclusion criteria to exclude articles that did not follow the specificity of this systematic review. Thereby, 13 articles were excluded (Supplemental Figure S1), and the remaining 30 were eligible to be analysed and applied 22 defined information criteria to extract the most relevant information (Supplemental Table S1).

## Results

### Bibliometric analysis

This section is focused on measuring scientific activity and mapping to present the overall picture for this review (Aria and Cuccurullo, 2017). The indicators of bibliometric analysis include annual scientific production, geographical distribution, journal distribution, most globally cited document, relevant authors and most frequent keywords.

#### Annual scientific production

From the set of selected documents, the trend in the number of publications addressing the life cycle approach to MBT units for municipal biowaste treatment over the last 5 years is shown in Supplemental Figure S2. Publications grew over time, with a slight decrease in 2022 but continued to increase in 2023. This shows a modest yet growing interest within the scientific community.

#### Geographical distribution

Concerning the number of studies conducted based on geographical locations, Italy had the highest number of studies (10), followed by the United States (5) and Germany (3) (Supplemental Figure S3). The other countries that produced the studies in this review had less than or equal to two studies. The European continent had the most significant publications, accounting for over 50% of all studies. Since most LCA studies concentrate on regions like Europe and Asia, there is a substantial research gap in areas like Africa, where the scarcity of reliable data presents a significant challenge.

#### Journal distribution

The studies under analysis were published in 19 journals, 7 of which present more than 1 study highlighted in this review. Notably, more than half of the studies reviewed were published in only seven journals, as illustrated in Supplemental Figure S4.

#### Keywords

The data generated by the Rstudio tool identified 96 keywords used by the authors of each article. The word cloud indicates greater prominence to the terms that appear most frequently in the selected articles (Supplemental Figure S5). The predominant terminologies employed by the authors in the articles are ‘anaerobic digestion’ and ‘life cycle assessment’. Subsequently, the terms ‘composting’, ‘food waste’ and ‘organic fraction of municipal solid waste’ followed in the frequency of usage.

#### Global citation

As for the most cited studies globally, Edwards et al. (2018) obtained, with a significant difference, the highest number of citations over time (106), followed by Lee et al. (2020) (60) and Mayer et al. (2020) (47) (Supplemental Figure S6).

### Qualitative and quantitative analysis

This section discusses the four phases of the life cycle approach and how it is considered in the selected articles, according to ISO 14044 reference parameters: goal and scope, inventory analysis, impact assessment and interpretation phase.

#### Goal and scope

The goal and scope is the first stage of the LCA, defined to be unambiguously articulated and aligned with the intended use of the information and should be adjusted throughout the study to ensure accuracy and relevance (ISO 14044, 2006). Due to the heterogeneity of each LCA, the scope differs depending on the specificity of each study. Most articles analysed defined the goal and scope intending to evaluate various waste management systems and indicate the optimal option (Supplemental Table S2). For such endeavours, they predominantly evaluated alternatives for organic waste management, mainly AD and composting. In contrast, less prevalent methodologies, such as waste-to-energy, gasification Fischer–Tropsch (GFT), hydrothermal carbonisation and anaerobic dynamic membrane bioreactors (AnDMBRs), were also found.

##### Feedstock type

**Figure 1.**
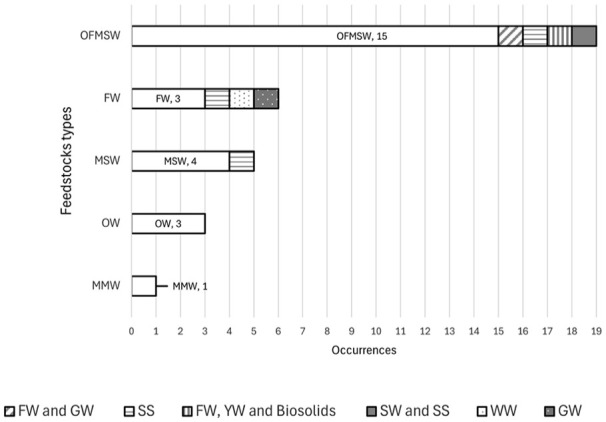
Feedstock used from the analysed studies. OFMSW: organic fraction municipal solid waste; FW: food waste; GW: garden waste; YW: yard waste; MSW: municipal solid waste; SS: sewage sludge; MMW: Mixed municipal waste; SW: slaughterhouse waste; OW: organic waste; WW: wood waste: WW.

Municipal biowaste is usually collected from various sources, including household waste, restaurants, offices, food processing plants, gardens and park maintenance (Baquero et al., 2022). The authors referred to the feedstock derived from municipal waste in their studies using various nomenclatures based on the specific type of waste and its characterisation. Organic fraction municipal solid waste (OFMSW) was the most used definition mutually exclusive (*n* = 15), followed by municipal solid waste (MSW) (*n* = 4), food waste (FW) (*n* = 3), organic waste (*n* = 3) and mixed municipal waste (*n* = 1) (Figure 1). Furthermore, some studies also evaluated the incorporation of FW and garden waste (GW) (*n* = 1), sewage sludge (SS) (*n* = 1), FW, yard waste (YW) and biosolids (*n* = 1) and sewage sludge and SS (*n* = 1) within OFMSW. Besides, FW was also analysed using SS (*n* = 1), WW (*n* = 1) and GW (*n* = 1). MSW also had one feedstock evaluated together, SS (*n* = 1).

Besides mixed municipal waste, some studies also evaluated the incorporation of SS, YW, GW, wood and slaughterhouse waste in treating urban organic waste.

##### Functional units

**Figure 2.**
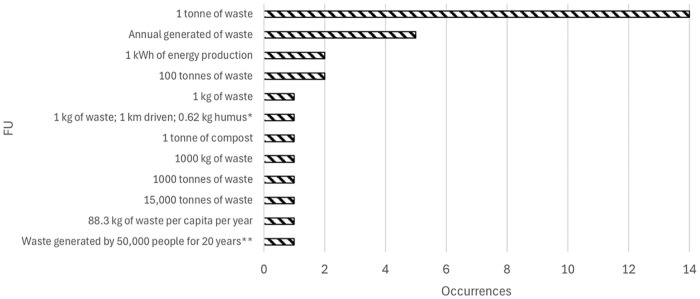
Functional unit (FU) used from the studies analysed. *One kilogram of waste and 1 km driven with a biogas-powered car and the production of 0.62 kg humus equivalents. **Waste generated by a municipality of 50,000 people in the United States for 20 years.

In the case of functional unit (FU), the predominant used unit was per tonne of waste treated, which stands out in the literature for the impacts of upstream waste (Figure 2). As the articles evaluated many waste treatment plants, several authors have based their analysis on the annual generated waste units due to the large amounts of waste these plants operate. Other authors have chosen specific units for their studies, such as ‘1 kg of waste and 1 km driven with a biogas-powered car and the production of 0.62 kg humus equivalents’, ‘88.3 kg of waste per capita per year’ and ‘waste generated by a municipality of 50,000 people in the US for 20 years’. The FU relies highly on each study’s scope; hence, the results’ heterogeneity. Only one author used 1 tonne of compost, which should represent the central product in LCA.

##### System boundaries

The system boundary defines the limits of the assessment, indicating which stages of the product’s life cycle are considered in the analysis. System boundary definitions according to ISO 14040 and 14044 (ISO 14040, 2008; ISO 14044, 2006) are cradle-to-grave, cradle-to-gate and gate-to-gate. Understanding these different system boundaries is crucial for interpreting the results of the waste management assessments conducted in the reviewed articles, as it determines which stages of the product’s life cycle are included and analysed for their environmental impacts.

**Figure 3.**
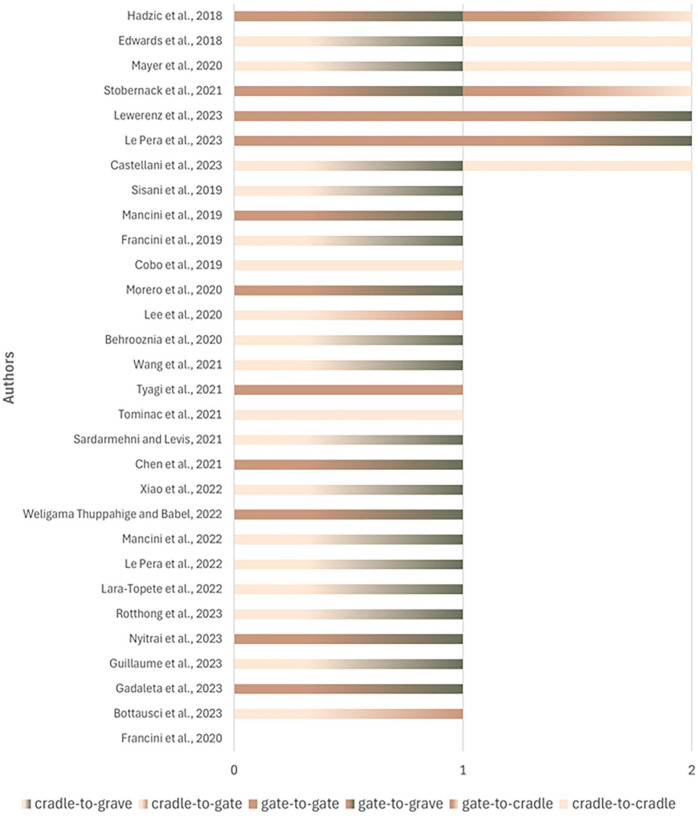
System boundaries defined for inventory analysis from the articles.

The assessed system boundaries predominantly represented cradle-to-grave (*n* = 14), with the second most used being gate-to-grave (*n* = 10) and the third cradle-to-cradle (*n* = 5) (Figure 3). Seven of the 30 articles analysed used 2 integrated concepts, cradle-to-grave plus cradle-to-cradle (*n* = 3), gate-to-gate plus gate-to-grave (*n* = 2), gate-to-grave plus gate-to-cradle (*n* = 2). Some authors who used the cradle-to-grave concepts included cradle-to-cradle in their analysis, expanding the system to a transition towards the circular economy (Castellani et al., 2023). In just one article, the explicit definition of the system employed was not provided (Francini et al., 2020).

#### Inventory analysis

##### Pre-treatment

The approach to waste collection or transport is contingent upon the particularities of each study, accounting for standard parameters, including vehicle types, fuel specifications, maximum load capacities, average travel distances and designated routes. In this review, six authors did not include waste collection and transportation data in their inventories (Table 1).

**Table 1.** Information on waste collection or transport methods and mechanical treatment or pre-treatment utilised in the studies.

Articles	Waste collection and transport	Mechanical treatment and pre-treatment
Edwards et al. (2018)	✓	Sorting, chipped
Hadzic et al. (2018)	✓	Rotary sieves
Cobo et al. (2019)	✓	Drum, magnet and Eddy current separator
Francini et al. (2019)	✓	Sorting, DF
Mancini et al. (2019)	✓	Chipper, electric screw mixer, disc scrubbing
Sisani et al. (2019)	x	Mechanical sorting
Behrooznia et al. (2020)	✓	Sorting
Francini et al. (2020)	✓	Shredder, electromagnet, rotating drum screen
Lee et al. (2020)	✓	Shredder
Mayer et al. (2020)	✓	Drying
Morero et al. (2020)	x	x
Chen et al. (2021)	x	Sorting, crushing, pulping, moisture-heat and three-phase separation
Sardarmehni and Levis (2021)	✓	Grinding, separation
Stobernack et al. (2021)	✓	OFMSW stored immediately. The following step removes impurities and structural material and mixes the substrate with water
Tominac et al. (2021)	✓	Screening, mixing, dewatering
Tyagi et al. (2021)	✓	Sorting, screening, shredder
Wang et al. (2021)	✓	Grinding, storage, equalisation
Lara-Topete et al. (2022)	✓	Manual separation, rotary drum, optic separators, magnetic separators, Foucault separators, RDF production
Le Pera et al. (2022)	✓	Drum, mixing
Mancini et al. (2022)	✓	I^a^
Weligama Thuppahige and Babel (2022)	x	x
Xiao et al. (2022)	✓	x
Bottausci et al. (2023)	✓	Shredding, mechanical shovels
Castellani et al. (2023)	✓	A bag opener, a magnetic separator, a disc sieve and a shredder
Gadaleta et al. (2023)	x	I^a^
Guillaume et al. (2023)	✓	x
Le Pera et al. (2023)	✓	Bag opener and a solid waste separator, shredder
Lewerenz et al. (2023)	✓	x
Nyitrai et al. (2023)	x	x
Rotthong et al. (2023)	✓	x

DF: dark fermentation; RDF: refuse-derived fuel; OFMSW: organic fraction municipal solid waste.

✓: Waste collection or transport included in the inventory analysis.

x: Not included.

a Inventory not available.

The predominant approach observed in the analysis of articles (*n* = 13) was using a singular type of truck for transportation, each associated with an average distance determined by the specificity of the context. Five articles incorporated data on collection and transport. However, the information was unavailable in the presented inventory. Additionally, five articles employed multiple types of trucks and varied distances to approach diverse scenarios and transfer stations between the collection point and treatment plant (Supplemental Table S3). Solely one investigation relied on literature-derived data concerning collection and transportation (Sardarmehni and Levis, 2021).

Pre-treatment methods are applied to the waste before further treatment or disposal. It includes details about sorting techniques, shredding, chipping, screening, grinding, mixing, dewatering and other mechanical processes to prepare the waste for subsequent treatment. Additionally, specific equipment is used in these processes, such as drum, sieves, magnetic separators, eddy current separators, shredders, disc screens, screw mixers and chipper machines (Table 1). Seven of the 30 articles did not include mechanical treatment or pre-treatment in their inventory analysis, and 2 authors either omitted consideration of these pre-treatment techniques and equipment or failed to provide them within the inventory data.

##### Biological processes

MBT is a strategy that combines the mechanical separation of recoverable materials with the biological stabilisation of organic matter for efficient management of MSW (Lara-Topete et al., 2023). AD and composting are biological stabilisation processes often combined with MBTs for energy and compost production (Kabaivanova et al., 2022).

Parameters related to composting practices adopted in each study include information such as the use of machinery, fossil and electricity consumption, emissions to air, water and soil and specific composting techniques like windrow composting, tunnel composting and forced aeration (Supplemental Table S4).

According to the results of this review, three authors did not detail or make available the inventory for composting and AD process (Supplemental Table S4). One author did not include the AD process in their inventory analysis, and six did not make any post-treatment.

According to the results, most of the articles consider inventory data on the use of machinery, energy consumption (fossil and electricity) and air, water and soil emissions (*n* = 27). Specific techniques during the composting process (windrow composting, tunnel composting and forced aeration) were used by around 16 authors (Supplemental Table S4).

Of the 26 studies that included the AD process, 3 did not provide detailed information or make their inventories available (Supplemental Table S4). Two authors included chemical inputs (FeCl_3_, NaOH and calcium hydroxide) (Supplemental Table S4). In addition, energy production data from the digestate was highlighted in six articles, one of them on the biogas combustion process, eight concerning the combined heat and power, two on produced biomethane and one utilised some filter to depurating (bio-scrubber) (Supplemental Table S4).

The post-treatment composting and AD processes conducted on the MBT unit represent an expansion of the base system. In this stage, the processed waste is finally directed towards disposal, as illustrated in Supplemental Table S4. Of the reviewed articles, 70% referred to compost application across various domains, including agriculture and landscaping. About 33% of the articles featured the utilisation of AD processes to facilitate the generation of either electrical or thermal energy, thereby fostering energy production from a renewable source. Additionally, 30% of the articles delineated the disposal of treated waste through conventional means, namely landfills or incineration. Of the reviewed literature, 13% denoted the mitigation and supplementation of fertilisers through compost utilisation, thus contributing to sustainable agricultural practices.

##### Gas measurements and estimates

In LCA studies, understanding gaseous emissions is fundamental to estimating the environmental impacts (Lara-Topete et al., 2023; Skaar et al., 2022). These data can be achieved through estimation or direct measurement of emissions. Estimation uses existing models and data to calculate emissions, while measurement involves directly collecting emissions data using instrumentation and monitoring methods, offering more accurate information (Lyu et al., 2021). In this context, only 7 articles made gaseous measurements and the rest (23) estimated them by other sources, including literature in general (12), Ecoinvent database (4), Intergovernmental Panel on Climate Change (IPCC) reports (3), software databases (3), theoretical models (3), unspecified references (3), national reports such as Australasian Unit Process LCI and Italian Greenhouse Gas Inventory Report (2), EMEP/EEA (1) and EPA (1) (Supplemental Figure S7).

During the inventory phase, the LCA research requires collecting data that must be obtained on-site, known as primary data, along with data that supplement the primary data, typically derived from literature published, databases and software, known as secondary data. Primary data on the processes is essential for LCA, as it reduces uncertainties and provides more accurate results (Jiang et al., 2020).

**Figure 4.**
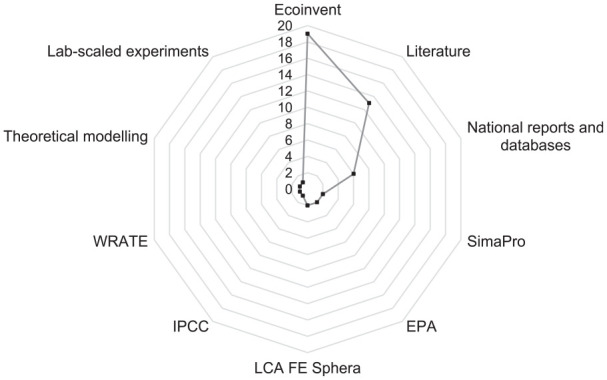
Sources from which the secondary data were taken.

Of the articles, 23 used primary and secondary data, 4 employed solely secondary data, while only 3 utilised exclusively primary data. Secondary data were obtained from the Ecoinvent database (19), published literature (13), national reports and databases (6) and five were gathered through software databases (SimaPro, LCA FE Sphera and WRATE) (Figure 4). The remaining data were derived from EPA sources (two), theoretical modelling such as mathematical formulas and equations (one), lab-scaled experiments (one) and the IPCC (one), comprising five occurrences.

#### Impact assessment

**Figure 5.**
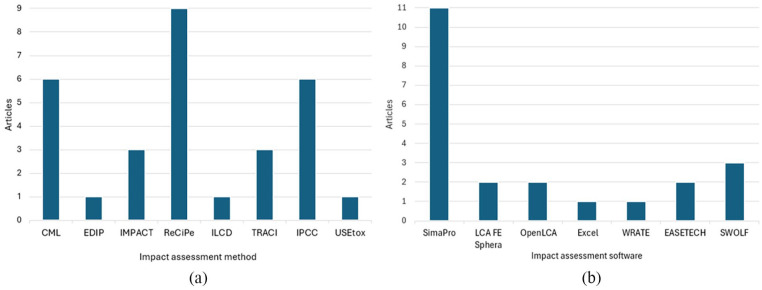
(a) Impact assessment method and (b) software most cited by the analysed studies.

During the life cycle impact assessment (LCIA) phase, characterisation methods convert inventory data into environmental impact assessment scores (Hauschild et al., 2013). Specialised LCA software is often employed to conduct impact assessment, streamlining inventory data processing, characterisation method application and analysis (Haque, 2020). The most used impact assessment method was ReCiPe (*n* = 9), CML and IPCC (*n* = 6), TRACI and Impact 2002+ (*n* = 3) and EDIP, ILCD and USEtox (*n* = 2) (Figure 5(a)). Two of the 30 articles used more than one characterisation method. Sisani et al (2019) used the methods ILCD 2011+ (midpoint) and IMPACT 2002+ (endpoint). Sardarmehni and Levis (2021) used the methods TRACI to calculate acidification, eutrophication and photochemical smog, IPCC 2013 to quantify 100-year GWP, whereas USEtox was used to determine ecotoxicity and human toxicity. Three of the 30 articles did not specify the methods used (Guillaume et al., 2023; Lewerenz et al., 2023; Tominac et al., 2021). Additionally, the predominantly used software were SimaPro (*n* = 11), SWOLF (*n* = 3), LCA FE Sphera, OpenLCA and Easetech (*n* = 2), Excel and WRATE (*n* = 1) (Figure 5(b)). Two of the 30 articles used 2 software for characterisation method application. Cobo et al. (2019) used Easetech for material flow analysis and LCA and SWOLF for LCC submodels. Tominac et al. (2021) used the SWOLF model to estimate environmental and cost indicators, and SimaPro modelled embedded impacts in the consumption of electricity to the production of nitrogen, phosphate (P_2_O_5_) and potash (K_2_O). Ten of the 30 articles did not specify which software they used (Chen et al., 2021; Francini et al., 2019, 2020; Lara-Topete et al., 2022; Le Pera et al., 2022, 2023; Lewerenz et al., 2023; Mancini et al., 2022; Nyitrai et al., 2023; Wang et al., 2021).

**Figure 6.**
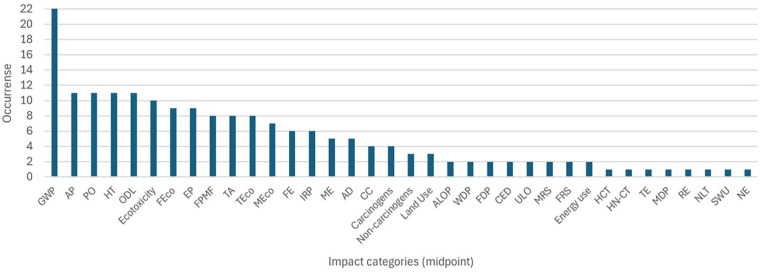
Impact categories (midpoint) cited by authors. GWHH: global warming human health; FPMF: fine particulate matter formation; HCT: human carcinogenic toxicity; HN-CT: human non-carcinogenic toxicity; GWTE: global warming terrestrial ecosystems; GWFE: global warming freshwater ecosystems; TA: terrestrial acidification; TE: terrestrial eutrophication; FE: freshwater eutrophication; ME: marine eutrophication; TEco: terrestrial ecotoxicity; FEco: freshwater ecotoxicity; MEco: marine ecotoxicity; GWP: global warming potential; AP: acidification potential; POP: photochemical oxidation potential; EP: eutrophication potential; HT: human toxicity; OLD: ozone layer depletion; AD: abiotic depletion; RD: re-source depletion; WL: waste landfill; CC: climate change; ALOP: agricultural land occupation potential; WDP: water depletion potential; FDP: fossil depletion potential; IRP: ionizing radiation potential; MDP: metal depletion potential; CED: cumulative energy demand; N-carcinogens: non-carcinogens; RE: respiratory effects; NLT: natural land transformation; ULO: urban land occupation; N-ReR: non-renewable and renewable; SWU: stressed water use; NE: nutrient enrichment; ER: energy resources; NEBR: net energy balance ratio; BCR: benefit cost ratio; LU: land use.

Another essential set of parameters to be considered in the LCIA phase relates to assessing environmental impacts. Characterisation factors and inventory data are mathematically combined to provide category indicator results, expressed in a unit standard to all contributions within the impact category (Hauschild et al., 2013). An extensive range of impact categories are applied in LCA. The GWP midpoint impact category was by far the most cited, twice more often cited than any other category (Figure 6).

The conventional way of quantifying climate change consequences involves employing the IPCC characterisation model, which includes parameters for calculating the GWP of various GHG emissions in kilograms of carbon dioxide equivalents (CO_2_ eq) (IPCC, 2014). These factors are provided for two different time frames, each with particular assumptions regarding the impact of methane. Advances in the underlying IPCC radiative heat forcing model lead to periodic revisions of characterisation factors, potentially introducing variability when comparing findings from older and more recent studies (Ebner et al., 2018).

Two of the 30 articles did not use midpoint environmental impact categories, as Tominac et al. (2021) considered only economic aspects (LCC) and Weligama Thuppahige and Babel (2022) estimated endpoint environmental impact categories (Human Health, Ecosystems and Resources).

**Figure 7.**
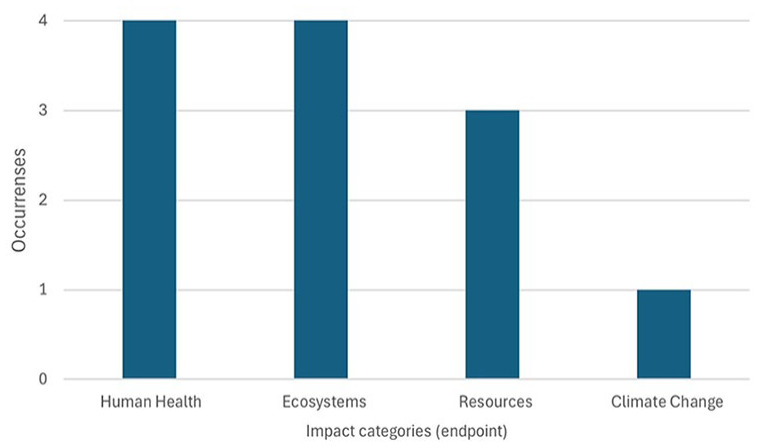
Impact categories (endpoint) cited by authors.

In the endpoint (damage impact) categories, Human Health and Ecosystems were the most cited (*n* = 4), Resources second (*n* = 3) and Climate Change (*n* = 1) (Figure 7). Only four articles generally employed endpoint indicators (Behrooznia et al., 2020; Gadaleta et al., 2023; Sisani et al., 2019; Weligama Thuppahige and Babel, 2022).

Integrating LCA and LCC offers a comprehensive approach to evaluating the sustainability of waste prevention, management and valorisation pathways and enables tracking of both environmental and economic impacts, providing valuable insights into sustainability (De Menna et al., 2018).

**Figure 8.**
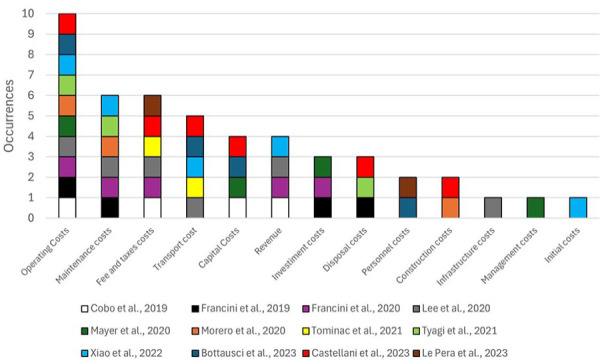
Main economic aspects cited by the authors that used LCC and their occurrences. LCC: life cycle costing.

In this systematic review, only 12 articles analysed applied the LCC approach in their studies. Operating costs were the most mentioned aspect (*n* = 10), followed by maintenance and fee and taxes costs (*n* = 6), tailed by transport cost (*n* = 5), capital costs and revenue (*n* = 4), investment and disposal costs (*n* = 3), personnel and construction costs (*n* = 2) and then infrastructure, management and initial costs were discussed one time (Figure 8).

#### Interpretation

In the results interpretation phase, data available from articles were used. Due to the wide dispersion of impact categories, only the GWP impact category was analysed as it was the most cited by the authors. Another contributing factor for this category selection was that such results share an equivalent indicator, and the emissions factors are very similar among the impact assessment methods (kg CO_2_ eq tonne^−1^ of biowaste).

**Figure 9.**
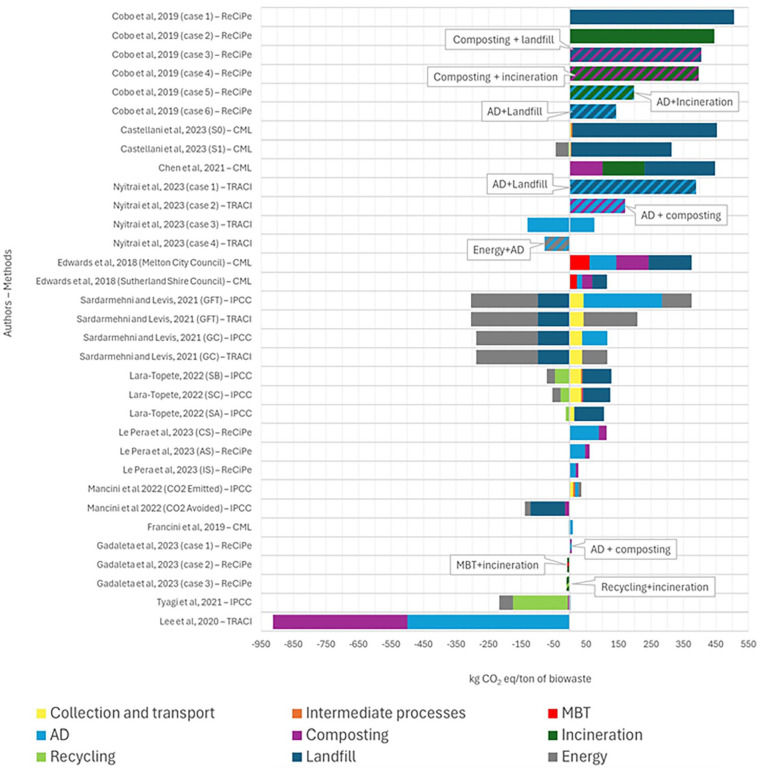
Absolute results for the GWP impact category by the biowaste management treatments. S0: MSW storage at the municipal collection centre, transportation to the mainland of Sicily and disposal into the sanitary landfill located on the same mainland; S1: plant constructions and operation, OFMSW treatment (AD and composting) and application of the compost on the island of Lampedusa. GFT: gasification Fischer–Tropsch; GC: gasification combustion; SA: current management scenario (landfill); SB: MT + industrial recycling + cement industry + landfill; SC: MT + industrial recycling + cement industry + composting + landfill; CS: current scenario (full-scale AD reactor and of a composting plant); AS: advanced scenario (AD and composting process a 3% reduced content of improper materials in separately collected FW); IS: ideal scenario (AD and composting with the total absence of unwanted materials in separately collected FW): GWP: global warming potential; AD: anaerobic digestion; OFMSW: organic fraction municipal solid waste; MSW: municipal solid waste; FW: food waste.

Cobo et al. (2019) compared six cases with four waste treatment processes and observed that landfills and incineration achieved the highest GWP estimates (Figure 9). Conversely, case 5 included incineration and case 6, an AD treatment followed by a landfill, had the lowest value (199 and 144 kg CO_2_ eq, respectively). Landfill and incineration treatments combined with composting had slightly lower GWP estimates (405 and 398 kg CO_2_ eq, respectively).

In Castellani et al. (2023), the transition from scenario S0 to S1 results in a 60% reduction in the net GWP impact. This improvement is primarily due to the introduction of a recycling process and shifting the electricity mix used in intermediate facilities, incorporating waste-to-energy sources. Additionally, avoiding landfill disposal is critical in reducing the environmental impact in S1 compared to S0 (Figure 9).

Chen et al. (2021) ranked the treatment scenarios regarding the GWP from the highest impact on landfilling to incineration and then composting (218, 129 and 101 kg CO_2_ eq tonne^−1^ of biowaste, respectively) (Figure 9). The higher contribution ratios were mainly due to the high energy consumption of the processing in the composting and incineration and the high emission concentrations of pollutants in the landfill.

Nyitrai et al. (2023) investigated novel high-rate AD technologies, including AnDMBRs. They found that all alternative management systems had lower net GWP impacts than the combination of landfill and AD because fugitive methane emissions accounted for 92% of the GWP burdens in this system (Figure 9). In the case of composting + AD, it had the second-highest GWP impact, mainly because fertiliser offsets only 6% of the total burden. The AD processes, which were complementary to the production and use of energy, had the highest net energy returns, avoiding using fossil fuel for electricity and thermal energy consumption. Co-management of food waste with SS proved superior to separate mono-digestion, avoiding costs associated with adding chemical additives and inoculums while improving energy recovery through efficient substrate conversion into methane. The study concluded that source-segregated and co-managed systems, with food waste entirely diverted from landfills, had a lower environmental impact than single and mixed waste streams sent to landfills.

The article from Edwards et al. (2018), which analysed two cases of study, got the most significant gross GWP impact for Melton City Council (375 kg CO_2_ eq tonne^−1^ of waste) compared with Sutherland Shire Council (115 kg CO_2_ eq tonne^−1^ of waste) (Figure 9). AD-based systems were shown to outperform composting-based systems significantly. However, the smallest GWP was found for AD for Sutherland Shire Council (17 kg CO_2_ eq tonne^−1^ of waste) and MBT for Melton City Council (61 kg CO_2_ eq tonne^−1^ of waste). The landfill was the disposal option that contributed the most to increasing GWP in both cases.

Sardarmehni and Levis (2021) investigated several emerging waste management scenarios derived from waste-to-energy, such as GFT and gasification combustion (GC) (Figure 9). They found that source separation of the OFMSW can improve digestate quality for AD and energy production but increase the impact of collection and transportation, particularly in low population density areas. GFT generates much energy but consumes it significantly, making it less favourable for compensation. In contrast, GC did not require such meticulous waste collection, and although its electricity generation was lower than that of GFT, it offset the gross impact, resulting in a net impact almost twice as small as that of the GFT without AD. Processing biological waste in anaerobic digesters made no difference in the GC scenarios but increased the net impact in the case of GFT.

For scenarios from Lara-Topete et al. (2022), scenarios B and C exhibit higher gross CO_2_ eq emissions than the current management system (SA), mainly due to fuel consumption in increased distances travelled by MSW transportation trucks and energy consumption by treatment plants (Figure 9). Despite higher gross GHG emissions in SB and SC, the balance between emitted CO_2_ eq and avoided emissions suggests that SA has the worst environmental performance regarding GHG emissions. Scenarios SB and SC reduced impact by about 45% and 59%, respectively. In SB and SC, material recycling at the intermediate facility and substituting petroleum coke with energy from refused-derived fuel are the primary sources of impact reduction.

The scenarios analysed in Le Pera et al. (2023) showed that the options with separately collected organic waste decreased CO_2_ eq emissions more than the current situation (CS) for both AD and composting processes (Figure 9). The scenario with reduced improper materials (advanced scenario (AS)) yielded environmental benefits due to decreased landfilling, reduced waste stabilisation and transport impacts. Additionally, it led to increased production of biomethane and compost, potentially replacing fossil fuels and chemical fertilisers, respectively. Compared to CS, the total GHG emissions decreased by 47% for AS and 23% for ideal scenario.

According to Mancini et al. (2022), approximately one-third of GHG emissions result from AD because of the direct emissions, with 8 kg CO_2_ eq tonne^−1^ of biowaste from AD and 3 kg CO_2_ eq tonne^−1^ of biowaste from biogas combustion. Transport activities add around 10 kg CO_2_ eq tonne^−1^ of biowaste. Electricity consumption and auxiliary material from intermediate facilities had a minor contribution at 9 and 5 kg CO_2_ eq tonne^−1^ of biowaste, respectively. Most of the avoided CO_2_ eq emissions come from diverting waste from landfills through bio-stabilisation during the composting process, which is attributed to various substitution activities and carbon sequestration. The second-largest contribution to reducing CO_2_ eq comes from renewable electricity production from biogas, and it is assumed that this renewable electricity reduces the national energy mix (Figure 9).

For Francini et al. (2019), the situation that included anaerobic co-digestion in AD-based systems reached negative values and reduced its net impact from 10 to 6 kg CO_2_ eq tonne^−1^ of biowaste because of the better overall energy recovery (Figure 9). This is due to the methanogenic phase’s improved specific gas production after the co-fermentation pre-treatment. The co-digestion process typically demonstrates better environmental performance than AD, and in this case, incorporating a preliminary stage of dark fermentation further enhanced the system’s environmental efficiency.

Gadaleta et al. (2023) evaluated disposal routes for packaging waste within organic, plastic and mixed waste streams. Significant differences in GWP were observed, with the organic waste stream scenario leading to a negative impact (GWP >0), whereas the recycling, MBT and integrated incineration scenarios resulted in environmental benefits (GWP <0) (Figure 9). When packaging waste was processed biologically alongside organic waste, the primary factor behind its poor performance was the failure to meet compost quality standards for agricultural use. This was due to the partial degradation of cellulose acetate in the packaging during AD and composting despite high biogas production. Recycling and mechanical treatment were crucial for improving outcomes, particularly in the scenario integrated with incineration.

The Tyagi et al. (2021) study showed a net impact of −215 kg CO_2_ eq tonne^−1^ of biowaste (Figure 9). The negative impact results from primary material recycling (79%) and exported electricity from refused-derived fuel (19%), which replaced grid and fossil-derived fuels. Recycled products from the MBT plant are anticipated to replace new products, reducing emissions from new raw production. The use of compost from the composting process and the high diversion of biowaste from landfills had a negative impact of about 5%, contributing 2% to the net negative impact. Positive emissions arise from diesel fuel consumption, inert material landfilling and intermediate facilities.

Lee et al. (2020) studied scenarios including windrow composting and AD-based systems (high-solids anaerobic co-digestion). Although both were beneficial, the AD-based system had the best performance (lowest net value), with a net value of −500 kg CO_2_ eq tonne^−1^ of biowaste. In contrast, the net value for the composting scenario was −413 kg CO_2_ eq tonne^−1^ of biowaste (Figure 9). Composting had a more significant influence on emissions, possibly due to the open operation of windrow composting, where waste is exposed to the atmosphere and fugitive emissions are not as well controlled as those in sealed anaerobic co-digestion reactors.

## Discussion

### Emissions factors and impact categories

A notable gap in the existing literature is the scarcity of in-situ gas measurements. Instead, authors commonly rely on estimates and assumptions gathered from secondary sources, thereby introducing potential inaccuracies. This reliance on referenced data may culminate in a proliferation of errors, resulting in both underestimation and overestimating outcomes. Moreover, the absence of standardised indicators and emission factors across different methods and methodologies further exacerbates this issue, leading to poor comparability among results.

The impact categories employed for organic waste treatments in an LCA are heavily influenced by decisions regarding the system boundaries and functional units (Serafini et al., 2023). In addition, significant variation among all impact categories can exist based on the organic waste feedstock properties, end-of-life treatment and implementation difficulty (Beigbeder et al., 2019; Florio et al., 2019).

The accountability of gas emissions and their associated impact categories is critical in assessing the environmental impacts of various waste treatment processes. For instance, nitrous oxide (N_2_O) emissions present challenges due to being hard to measure accurately and often leading to substantial uncertainties or errors (Mayer et al., 2020). Despite their low emission rates during composting, digestate spreading and incineration, the high impact factor of N_2_O emissions over a 100-year timeframe underscores their significant environmental ramifications.

In AD systems combined with composting, particulate matter emissions are linked to nitrogen oxide (NOx) and sulphur dioxide (SO_2_) released during biogas combustion, as well as ammonia emissions from digestate composting (Sardarmehni and Levis, 2021). These emissions contribute directly to the particulate matter formation impact category, reaffirming the need to carefully assess waste treatment biological systems’ environmental impact.

Acidification, eutrophication and particulate matter emissions predominantly arise from the composting process, with ammonia emissions and nitrate leaching exacerbating these impacts, particularly regarding eutrophication (Guillaume et al., 2023). The photochemical oxidation potential (POP) exhibit a notable impact, primarily attributed to the elevated volatile organic compound emissions during composting compared to landfill emissions. Additionally, biological treatment plants, particularly those lacking gaseous emissions treatment, significantly contribute to AP, with ammonia and electricity consumption being the primary contributors (Colón et al., 2015).

Nitrogen and phosphorus compounds released from landfills are identified as the primary drivers of EP, emphasising the significant impact of non-source-separated organic waste in MSW (Colón et al., 2015). Additionally, emissions from landfilling and incineration processes significantly contribute to climate change impacts, with landfilling emitting methane (CH_4_) and incineration, thus affecting the resource use of fossil fuels (Guillaume et al., 2023). Moreover, emissions from organic decomposition and burning of fuels during waste treatment processes account for a substantial portion of total emissions (Tominac et al., 2021).

Furthermore, ammonia (NH_3_), non-methane volatile organic compounds and hydrogen fluoride emissions are significant contributors to primary air pollutants and particulate matter, with emissions from composting and digestion processes dominating the impact scores across various treatment options (Lewerenz et al., 2023). Similarly, waste collection and transportation activities significantly contribute to ecotoxicity impacts due to high emissions of heavy metals from vehicle operations (Lee et al., 2020). In addition, the collection and sorting are excluded because of their minimal contribution (less than 5%) to the overall waste management life cycle (Nyitrai et al., 2023).

Additionally, sulphur and nitrogen emissions from human activities, such as NOx and ammonia, are primary contributors to atmospheric pollution, as measured by AP (Chen et al., 2021). Moreover, OLD remains a significant environmental concern, primarily attributed to the production and release of chlorofluorocarbons, emphasising the need for stringent measures to mitigate emissions contributing to these impacts (Behrooznia et al., 2020).

### Fertiliser substitution

Discussing the environmental impacts of fertiliser substitution using digestate and compost in agriculture deserves several considerations. Firstly, while substituting mineral fertiliser with organic alternatives like digestate and compost may lead to a slight offset in specific impact categories, it presents challenges related to its heavy metal contents. Heavy metals, such as Cd, Pb, Zn and As, released after the deployment of the digestate significantly contribute to human toxicity and ecotoxicity impacts (Mayer et al., 2020).

Conversely, the retention of phosphorus and potassium in ash during gasification offers potential benefits for fertilising purposes (Stobernack et al., 2021). However, the positive net GWP associated with spreading solid digestate on agricultural land, despite replacing mineral fertilisers, raises concerns about the environmental implications of this practice. Additionally, the lower availability of nitrogen in compost than in mineral fertiliser requires adjustments using mineral fertiliser equivalent factors to ensure adequate nutrient supply to plants (Sardarmehni et al., 2021).

Applying fertilisers to the soil may induce run-off of nitrogen and phosphorus into fresh and marine waters, contributing to the eutrophication of aquatic environments (Le Pera et al., 2023). Therefore, not using compost has a minor impact on the freshwater and marine water categories. However, the leaching of nitrogen and phosphorus from fertiliser compost is lower than that of chemical fertilisers. The primary avoided impacts due to compost use are caused by peat and nutrient substitution of nitrogen, phosphorus and potassium contained in the produced compost. Furthermore, avoiding chemical fertiliser production reduces significantly ecotoxicity, resource use of minerals and metals and water consumption (Guillaume et al., 2023).

The predominance of insoluble forms of inorganic nutrients in composted products reduces nutrient loss through leaching, highlighting the potential environmental benefits of compost as a soil amendment. However, carefully considering heavy metal emissions, nutrient availability and overall environmental impacts is essential in assessing the sustainability of using organic alternatives to fertiliser in agriculture (Hadzic et al., 2018).

### Relevant municipal waste treatment technologies

The discussion surrounding waste management methods, particularly incineration, landfilling, AD and composting, reveals contrasting environmental impacts and trade-offs associated with each approach. The incineration of pre-dried organic waste has led to higher environmental impacts and energy production costs, primarily due to increased energy consumption, which raises costs and may release volatile organic compounds and other atmospheric pollutants (Mayer et al., 2020). Additionally, the combination of AD with the incineration system exhibits the highest overall impact among the studied methods, suggesting its environmental drawbacks.

Landfill impacts significantly on GWP, OLD and POP. The generation of CH_4_ emissions during landfilling poses a primary environmental concern, underlining the urgency for improved landfill gas collection, combustion and energy production systems to mitigate these adverse effects (Escamilla-Alvarado et al., 2017). Six of the 13 articles addressed landfills’ GWP. In some studies, landfills’ higher impact may be partially attributable to the omission of energy recovery from biogas in these studies’ inventories or to its insufficient production to mitigate the GWP (Castellani et al., 2023; Chen et al., 2021; Cobo et al., 2019; Edwards et al., 2018; Lara-Topete et al., 2022; Tyagi et al., 2021).

On the other hand, the AD process demonstrates several environmental benefits, including increased carbon chain disintegration, water resource conservation and the generation of renewable biogas. The production of biogas through AD and the composting of digestates contributes to environmental load credits, particularly by preventing nitrogen release and promoting the reuse of organic matter as a fertiliser (Behrooznia et al., 2020).

Despite the favourable environmental profile of AD compared to composting, the latter remains an attractive and cost-effective method for increasing soil organic content and enhancing soil fertility. Separating the organic fraction from MSW can result in higher-quality feedstock for AD, increasing the efficiency of biogas. In addition, biogas purification methods for biomethane are crucial for increasing green energy efficiency and mitigating environmental impacts (Sardarmehni and Levis, 2021).

In conclusion, while each waste management method offers unique advantages and challenges, the findings of LCA studies underscore the need for a comprehensive assessment of environmental impacts to inform sustainable waste management practices. When comparing waste management systems, evaluating the net energy demand is a more effective indicator than simply measuring the amount of waste recovered for energy or the energy produced by valorisation technologies. This approach underscores the importance of assessing waste management practices’ overall efficiency and sustainability.

### Biowaste source-separated versus mechanical separation

Biowaste is often collected in undifferentiated containers, where various types of wastes are deposited, regardless of their nature. Many of these wastes exhibit high levels of biological and heavy metals contamination or contain significant amounts of inert material that hinder the efficiency of biological degradation processes (Kupper et al., 2014).

The efficiency of household sorting plays a crucial role in the effectiveness of waste management systems. If biowaste household sorting efficiency were to improve to the levels seen in plastic, metal and paper recycling, the waste diversion rate would likely increase significantly (Perrin and Barton, 2001). Enhanced sorting efficiency would lead to lower contamination levels, thereby improving the quality of the separated waste streams. Furthermore, the selective collection of organic fractions for dedicated biological treatment can reduce 14% and 35% of MSW discharged in landfills and incinerated, respectively (Sisani et al., 2019).

The MBT system, which handles mixed waste streams, including mechanically separated organic fractions of municipal solid waste (ms-OFMSW), can often compromise the recycling scenarios. The MBT system processes a mixture of FW and other types of waste. After the aerobic stabilisation, the application of compost results in a more significant amount of heavy metals and other contaminants, as these are present in higher concentrations in ms-OFMSW when compared to source-separated FW (Edwards et al., 2017). This issue is particularly pertinent when considering the circular economy, which aims to utilise resources at their highest utility while minimising waste.

Conversely, separating biowaste at the source significantly raises the costs and emissions related to waste collection, especially in areas with low population densities (Sardarmehni and Levis, 2021). While collecting source-separated biowaste enhances compost quality, it may not significantly improve the system’s overall environmental performance. This is because the energy recovered from source-separated biowaste may not sufficiently balance the environmental impacts of the energy consumed during its transportation.

### Limitations of the LCA approach

LCAs primary limitations are the assumptions made during modelling, particularly in substituting waste-derived products, which are a significant source of uncertainty and can influence the outcomes (Antelava et al., 2019). The lack of region-specific data and the exclusion of socio-economic aspects may limit the applicability and accuracy of the analyses. Insufficient documentation and a lack of clear justifications for substitution choices can undermine the credibility of LCA studies (Dastjerdi et al., 2021; Weidema, 2019). Many studies do not specify system boundaries or the functional units used, hampering comparisons between studies. Judging from the existent set of articles using LCA to assess biowaste management, the lack of global standardisation and clarity makes it difficult to compare scenarios and ensure consistency in results.

### European policies and statements

Biowaste management is crucial to the European Union (EU) circular economy strategy. Several policies and initiatives have been implemented to promote the recovery and sustainable treatment of biowaste. To achieve their goals, the EU adopted the Landfill Directive (1999/31/EC) to divert biowaste from landfills to recycling and recovery, supported by the Renewable Energy Directive (2018/2001/EU) for renewable energy use (biogas and biomethane) and the Fertilizing Products Regulation (EU) 2019/1009) to ensure safe organic fertiliser standards (European Commission, 1999).

European legislation promotes organic treatment and recycling as central themes, with technologies such as AD and composting widely used (Javadzadeh and Hamedeyaz, 2014). The production of biomethane from organic waste is a sustainable alternative to fossil methane (Araya, 2018). The compost used as soil conditioners should follow regulations based on the European directive to control the quality of composts used in agriculture, which helps ensure use safety (Collivignarelli et al., 2019). More robust quality standards for urban waste composts are crucial to promoting biological agriculture, avoiding market rejection and increasing consumer confidence (Chelinho et al., 2019; De Corato, 2020; Prasad and Foster, 2023).

Separate organic waste collection is promoted in certain European countries, but its effectiveness varies widely depending on public participation (Schüch et al., 2019). Targeted information campaigns and the strategic location of containers can increase public participation and reduce the contamination of collected waste (Slavík et al., 2018). Several technological, financial and regulatory challenges hinder the valorisation of organic waste (Siegfried et al., 2023; Suárez et al., 2023). Hence, innovative interaction between science and policymaking is necessary to understand the complexity of biowaste problems, formulate better regulations and overcome challenges to promote sustainable management (Duquennoi and Martinez, 2022).

## Final remarks

The publication of LCA studies on municipal biowaste treatment processes has continued to grow over the past 5 years, with European countries leading in the number of publications in this area. The reviewed studies exhibited heterogeneity in system construction and data collection methodologies. These methodological divergences create challenges in data benchmarking. The variety in feedstock usage, which often combines municipal waste with other materials, further contributes to this ambiguity.

Among the functional units used, there was a consensus on the general understanding of using the mass input of waste. However, from a product perspective, the compost’s mass, or any other usable output, would be the most appropriate functional unit. Most differences and gaps were observed in expanding system boundaries, ranging from a single life cycle stage (gate-to-gate) to whole circular approaches (cradle-to-cradle). Few studies have provided analyses of MBT alone. However, most articles present aggregated scenarios, addressing multiple life cycle stages, hindering the interpretation of individual process contributions. Using different emission factors and impact category indicators based on various methods also limits the comparison of results. Additionally, the reliance on secondary data instead of local data and field experiments is a common source of uncertainty.

Organic waste treatment systems based on mechanical and biological processes, AD and composting may provide more significant benefits due to the mechanical separation of materials, energy production and the replacement of synthetic fertilisers. In these processes, the selective collection of organic waste would enhance compost’s agronomic quality and economic aspects. However, it could also increase the burdens and emissions associated with collection and transportation.

While this study concentrated solely on environmental aspects, future research should incorporate social and economic assessments alongside the life cycle approach to achieve a holistic perspective on waste management planning.

## Supplemental Material

sj-docx-1-wmr-10.1177_0734242X251326866 – Supplemental material for Life cycle approach as a tool for assessing municipal biowaste treatment units: A systematic review Supplemental material, sj-docx-1-wmr-10.1177_0734242X251326866 for Life cycle approach as a tool for assessing municipal biowaste treatment units: A systematic review by Laís Fabiana Serafini, Paulo José Gomes Monteiro Praça, Fernando González-Andrés and Artur Gonçalves in Waste Management & Research
