# Supplementary material for: Life cycle approach as a tool for assessing municipal biowaste treatment units: A systematic review
Source: Waste Manag Res. 2025 Mar 27;43(10):1509–24. doi: 10.1177/0734242X251326866 (PMC12476467; doi:10.1177/0734242X251326866)
Supplement: sj-docx-1-wmr-10.1177_0734242X251326866 – Supplemental material for Life cycle approach as a tool for assessing municipal biowaste treatment units: A systematic review [file sj-docx-1-wmr-10.1177_0734242X251326866.docx]

Life cycle approach as a tool for assessing municipal biowaste treatment units': a systematic review

SUPPLEMENTARY INFORMATION


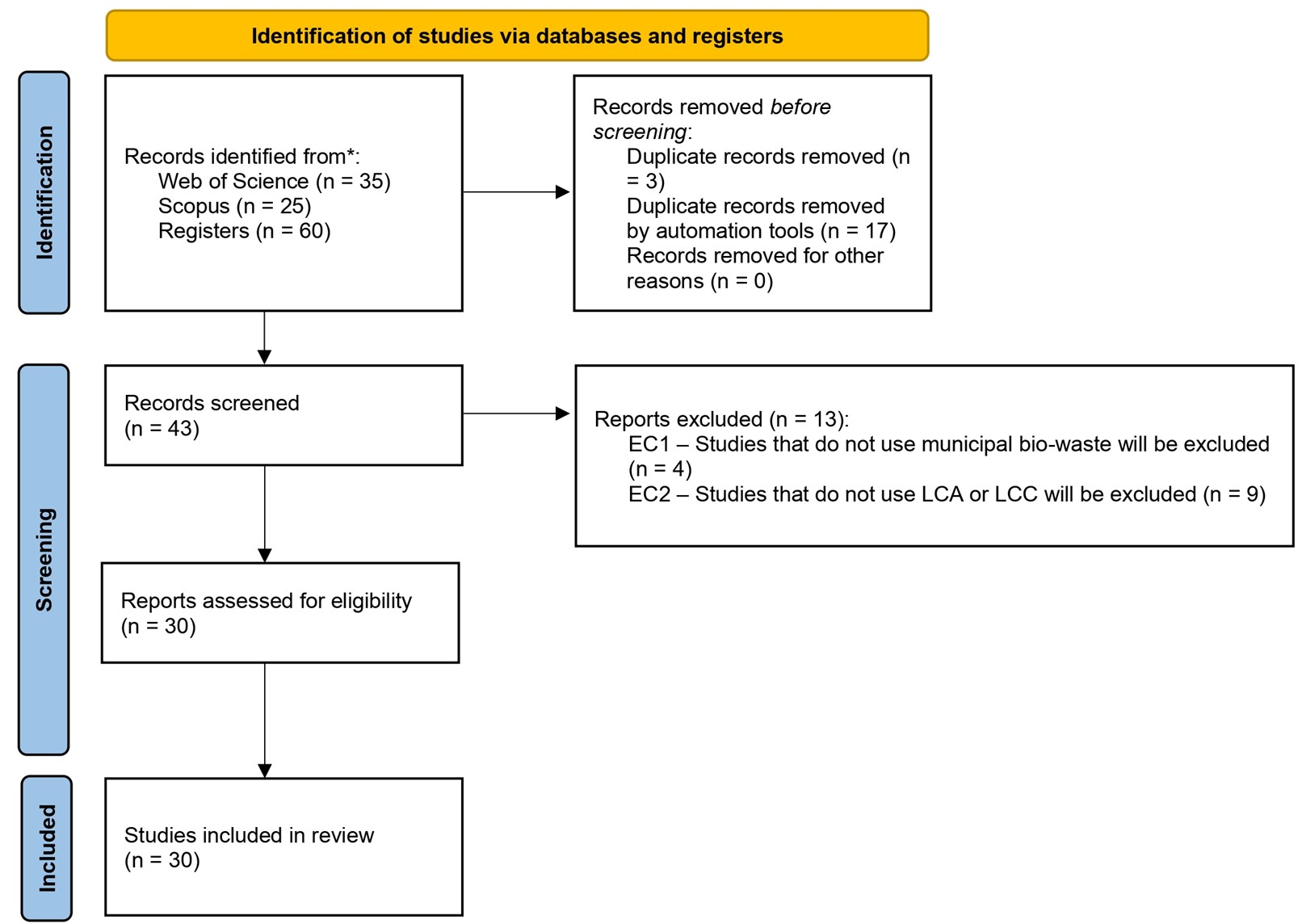


Figure S1: PRISMA 2020 flow diagram for new systematic reviews, which included searches of databases and registers only

Table S1: Information Extraction Criteria applied in this systematic review.

| **Field** | **Content Selection** |
| --- | --- |
| Goal and scope | Specific to each study |
| Feedstock type | Biowaste; Municipal organic waste; Urban biodegradable waste; Residential waste; Organic fraction municipal solid waste (OFMSW); Others |
| Functional unit | Tonne of waste; annual generated of waste; kWh of energy production; tonne of compost; kg of waste per capita per year; km driven |
| System boundary | Cradle-to-grave; Cradle-to-gate; Gate-to-gate; Gate-to-grave; Gate-to-cradle; Cradle-to-cradle; Bin-to-cradle |
| Waste collection and transport | Vehicle types; consumption and distances travelled averages |
| Mechanical treatment and pre-treatment | Sorting; Screening; Grinding; Drying; Dehydration; Dewatering; Others |
| Composting | Use of machinery; Fossil energy consumption; Electricity consumption; Water consumption; Forced aeration; Air emissions; Water emissions; Soil emissions; Leachates; Others |
| Anaerobic digestion | Use of machinery; Fossil energy consumption; Electricity consumption; Water consumption; Power utilization; Air emissions; Water emissions; Soil emissions; Leachates; Others |
| Post-treatment | Landfill; WtE; Incineration; Compost application; Power utilization; Others ; No |
| Gas emissions measurements | Yes; No |
| Gas emissions estimation | Literature; Ecoinvent; Intergovernmental Panel on Climate Change (IPCC); Software; Modelling; National reports, European Monitoring and Evaluation Programme (EMEP)/ European Environment Agency (EEA); Environmental Protection Agency (EPA) |
| Which impact assessment method was used | CML-IA; EDIP 2003; EPD 2013; EPS 2000; IMPACT 2002+; ReCiPe; ILCD 2011; TRACI; IPCC 2013; lime 2; Eco-Indicator |
| Impact assessment software | SimaPro; LCA FE Sphera; OpenLCA; Umberto; Excel; WRATE; EASETECH; LACSD; EASEWASTE; STAN; TOTAL |
| Impact category (midpoint) | Global warming Human health (GWHH); Fine particulate Matter formation (FPMF); Human carcinogenic toxicity (HCT); Human non-carcinogenic toxicity (HN-CT); Global Warming Terrestrial Ecosystems (GWTE); Global warming Freshwater ecosystems (GWFE); Terrestrial Acidification (TA); Terrestrial Eutrophication (TE); Freshwater Eutrophication (FE); Marine Eutrophication (ME); Terrestrial Ecotoxicity (TEco);  Freshwater Ecotoxicity (FEco); Marine Ecotoxicity (MEco); Global Warming Potential (GWP); Acidification Potential (AP); Photochemical Oxidation (PO); Eutrophication Potential (EP); Human Toxicity (HT); Ozone Layer Depletion (ODL); Abiotic Depletion (AD); Resource Depletion (RD); Waste Landfill (WL); Climate Change (CC); Agricultural Land Occupation Potential (ALOP); Water Depletion Potential (WDP); Fossil Depletion Potential (FDP); Ionizing Radiation Potential (IRP); Metal Depletion Potential (MDP); Cumulative Energy Demand (CED); Smog; Carcinogens; Non-carcinogens; Respiratory Effects (RE); Ecotoxicity; Natural Land Transformation (NLT); Urban Land Occupation (ULO); Non-Renewable and Renewable (N-ReR); Stressed Water Use (SWU); Nutrient Enrichment (NE); Energy Resources (ER); Net Energy Balance Ratio (NEBR); Benefit Cost Ratio (BCR); Land Use (LU); Mineral resource scarcity (MRS); Fossil resource scarcity (FRS); Energy Use (EU); Others |
| Damage category (endpoint) | Human health (HH); Ecosystems; Resources; Climate Change |
| Primary data (Inventory) | Yes; No |
| Secondary data (Inventory) | Ecoinvent; Literature; National reports and databases; SimaPro; EPA; LCA FE Sphera; IPCC; Waste and Resources Assessment Tool for the Environment (WRATE); Theoretical modelling; Lab-scaled experiments |
| Was there any application of techniques/ scenarios to improve the treatment? | Yes; No |
| Was applied LCC approach | Yes; No |
| LCC economic impacts | Inicial cost; Investment cost; Capital cost; Service cost; Transport cost; Preventative maintenance cost; Management cost; Operating cost; Disposal cost; Others |


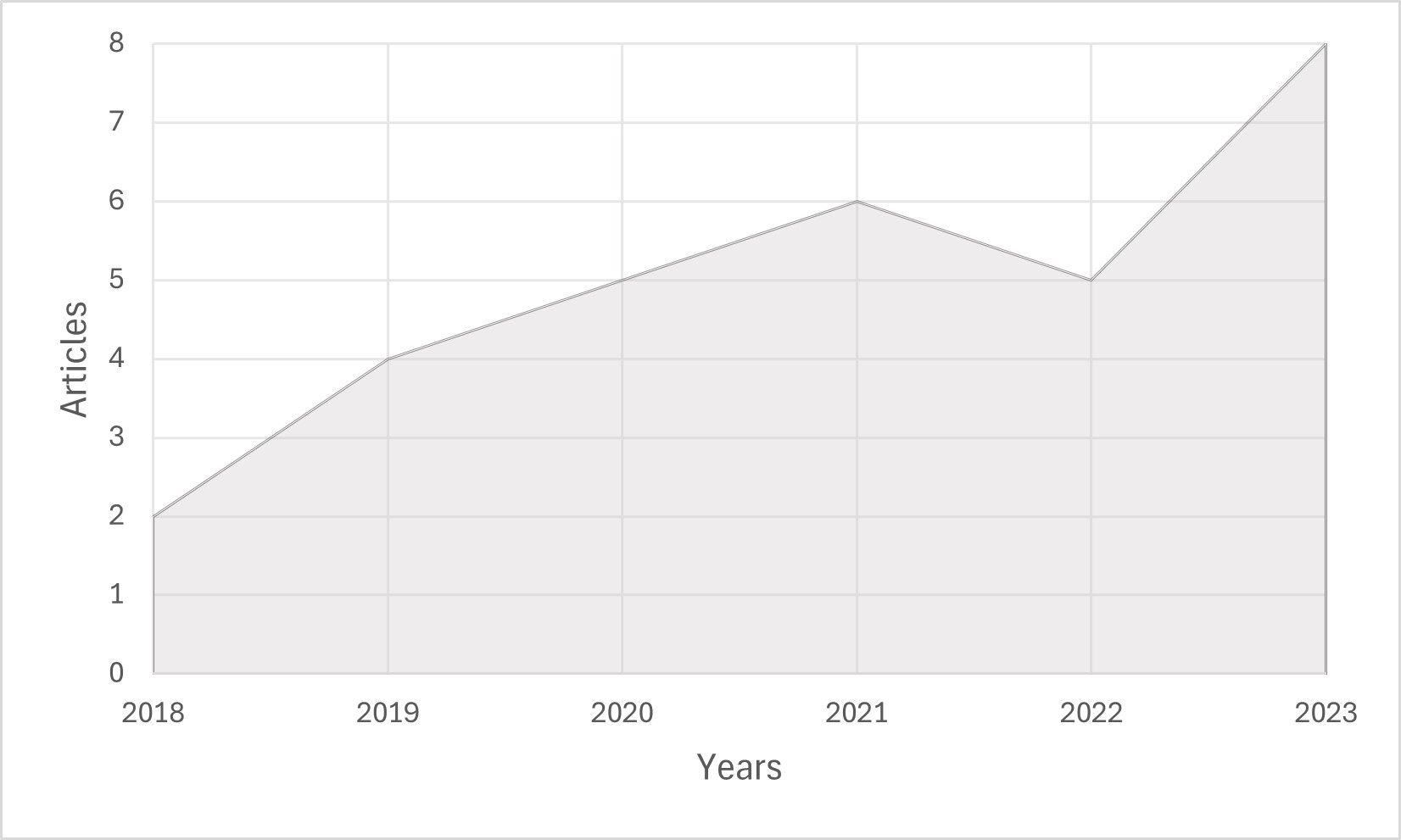


Figure S2: Annual scientific production


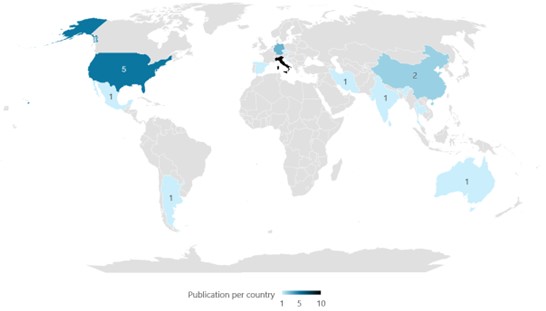


Figure S3: Countries' scientific production


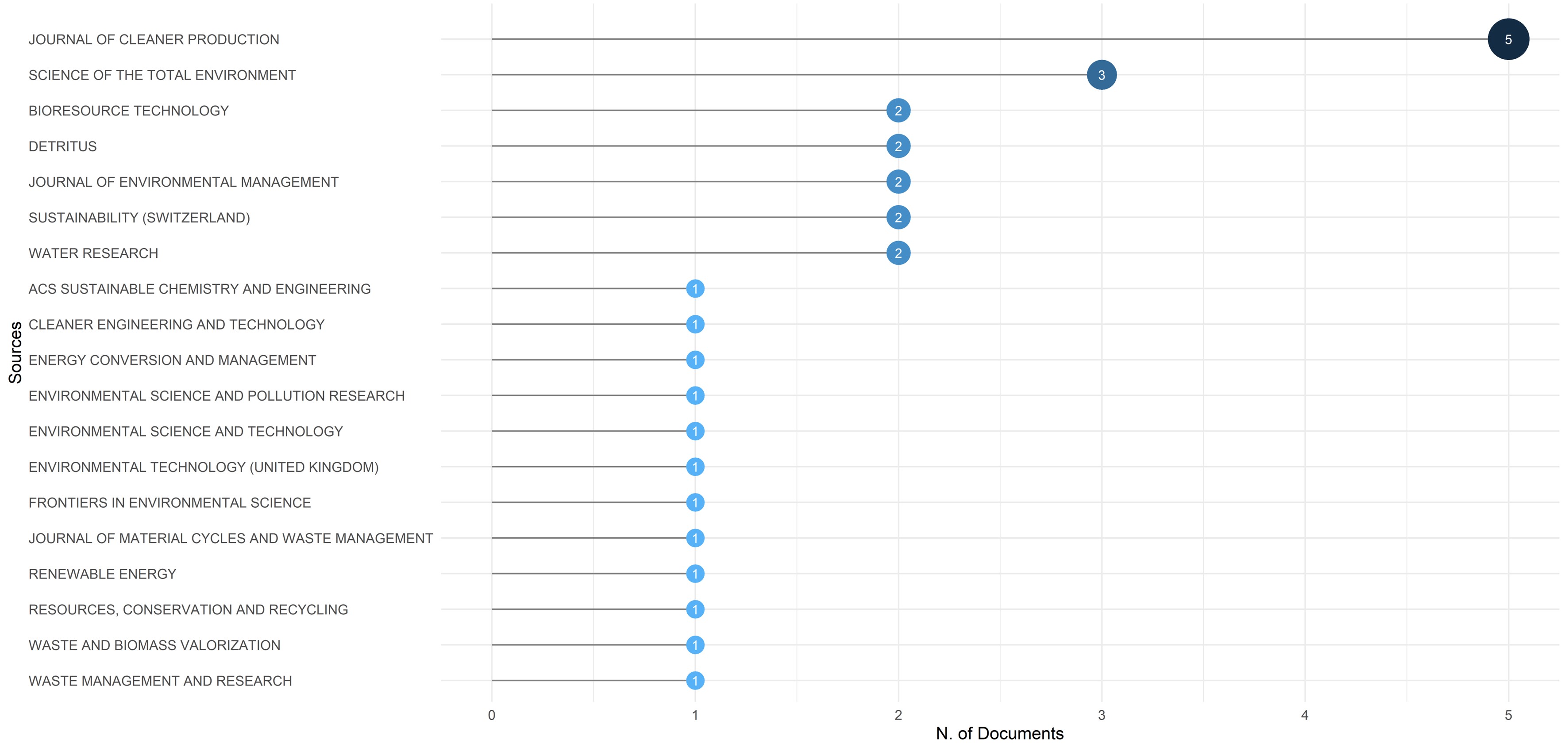


Figure S4: Distribution of reviewed studies in the scientific journals.


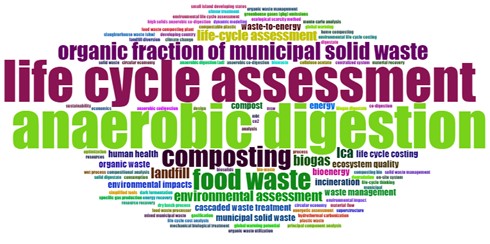


Figure S5: Word cloud from 96 keywords contained in the reviewed studies.


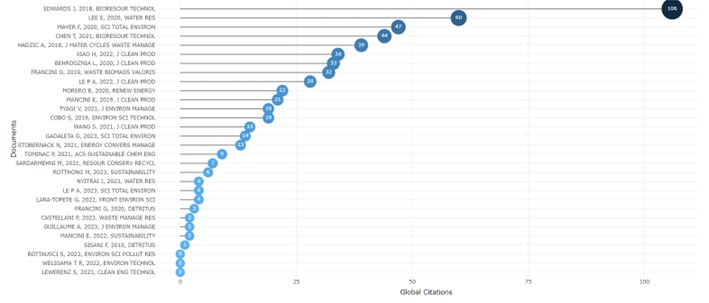


Figure S6: Globally cited studies.

Table S2: Goal and scope definition of the articles analysed.

| Articles | Goal and scope |
| --- | --- |
| (Edwards et al., 2018) | Provide decision-makers with an analysis to determine the most environmentally friendly waste management system for their waste catchment. |
| (Hadzic et al., 2018) | To determine the environmental performance of the existing waste management system from a Life-cycle perspective and to compare it with the sustainable system. |
| (Cobo et al., 2019) | Recovery of nutrients from organic waste (OW). |
| (Francini et al., 2019) | From an environmental and economic point of view, compare the treatment scenarios for SS-OFMSW (sewage sludge - organic fraction municipal solid waste) and SS. |
| (Mancini et al., 2019) | Identify the environmental hotspots of two OFMSW treatment options for a composting plant (without and with an anaerobic digestion plant) |
| (Sisani et al., 2019) | Examine the impact of increased materials and energy recovery from waste on human health and ecosystem quality. |
| (Behrooznia et al., 2020) | Evaluate the environmental impacts of MSW's (municipal solid waste) composting and anaerobic digestion (AD) process |
| (Francini et al., 2020) | Analyse two possibilities for the anaerobic treatment of SS-OFMSW considering its costs, primary energy used and CO_2_ equivalent emissions. |
| (Lee et al., 2020) | Assess the environmental impacts and benefits of High-Solids Anaerobic co-digestion) (HS-AcD) and compare its environmental impacts and benefits with different organic waste management strategies (composting, incineration, and landfilling). |
| (Mayer et al., 2020) | Determine which conventionally practised treatment paths are viable from an environmental point of view. |
| (Morero et al., 2020) | Determine the environmental impacts associated with the results of the waste management system. |
| (Chen et al., 2021) | Quantify the impacts of different solid digestate treatment methods and identify a feasible strategy for the improvement of the biogas system of the FW (food waste). |
| (Sardarmehni and Levis, 2021) | To compare the life-cycle impacts associated with landfilling, mass burn WTE (waste-to-energy), gasification and syngas combustion, GFT (gasification Fischer–Tropsch) to fuel, AC (aerobic composting), and AD. |
| (Stobernack et al., 2021) | Quantify the GWP of the HTC (hydrothermal carbonisation) process compared to the current base case ("AD + comp"). |
| (Tominac et al., 2021) | Use LCA methods to estimate environmental indicators and optimisation techniques to compare different MOW (municipal organic waste) diversion strategies. |
| (Tyagi et al., 2021) | Quantify the MBT plant's greenhouse gas (GHG) emissions from treating municipal solid waste (MSW). |
| (Wang et al., 2021) | Estimate the energetic performance and quantify the global warming impacts of treating SHW (Slaughterhouse waste) through AD and compare it with alternative treatments. |
| (Lara-Topete et al., 2022) | Compare the environmental performance of the current vs. two potential MSW management scenarios. |
| (Le Pera et al., 2022) | Quantify and compare the environmental impacts of the production system of two types of compost, one obtained from OFMSW and the other from digestate derived by AD of OFMSW |
| (Mancini et al., 2022) | To test the "CO2mpost" tool to analyse its strengths and weaknesses based on sector-specific literature and to draw more general indications for carbon footprint practitioners. |
| (Weligama Thuppahige e Babel, 2022) | Compare the environmental impacts of composting and AD from OFMSW management. |
| (Xiao et al., 2022) | Assess the environmental and economic impacts of AD-based FW treatment associated with biogas digestate (solid) disposal. |
| (Bottausci et al., 2023) | Analyse whether plastics in biowaste affect the final compost quality and generate negative environmental and economic consequences. |
| (Castellani et al., 2023) | Evaluate on-site treatment plants' environmental and economic benefits for valorising municipal solid waste's organic fraction. |
| (Gadaleta et al., 2023) | Quantify the environmental performances of the leading disposal routes for cellulose acetate (CA) packaging waste. |
| (Guillaume et al., 2023) | Environmentally assess current biowaste management strategies to identify critical parameters and to suggest improvements at a municipal level. |
| (Le Pera et al., 2023) | Quantify the environmental impacts of improper materials on the AD and composting industrial processes of SC-FW. |
| (Lewerenz et al., 2023) | Expand, update and evaluate the current and OFMSW treatment options (disposal perspective of biowaste treatment). |
| (Nyitrai et al., 2023) | Evaluate alternative food waste management systems and get improvements by adopting new management strategies, such as the AnDMBR system. |
| (Rotthong et al., 2023) | Assess the environmental impacts of different municipal organic waste management systems. |

Table S3: Detailed information on waste collection, transport, and mechanical treatment utilised in the studies.

| Articles | Waste collection and transport | Mechanical Treatment and Pre-treatment |
| --- | --- | --- |
| (Edwards et al., 2018) | BAU (Business-as-usual): Garbage (with FW) waste is collected weekly, and Garden waste is collected fortnightly. ACOD (Anaerobic co-digestion): FW is collected weekly, and the garbage bin is collected fortnightly. COMP (Centralised composting): organics (with FW) are collected weekly, and garbage bins are collected fortnightly. HCOMP (home composting): non-organic garbage is collected fortnightly at the curbside, and large garden waste items are collected monthly and chipped. MBT (Mechanical biological treatment): has a collection regime like in BAU with FW and other garbage discarded into one bin. | Sorting, chipped |
| (Hadzic et al., 2018) | 10 tons, EURO 3, 1.57 l/t | Rotary sieves |
| (Cobo et al., 2019) | Average distance between municipalities to the transfer station (24.7 km) and from the transfer station to the treatment plant (21.6 km). | Drum, magnet and Eddy current separator |
| (Francini et al., 2019) | Door-to-door collection. Transportation of the digestate exiting by the dewatering process to the aerobic bio-stabilisation plant was considered, assuming a distance of 270 km. The transport is a freight lorry transport with 16–32 t of capacity (Ecoinvent) | Sorting, Dark Fermentation (DF.) |
| (Mancini et al., 2019) | Vehicles used for compost distribution can carry a maximum load of 10–11 tonnes. All outbound trips are carried out at full load whilst they return empty. | Chipper, electric screw mixer, disk scrubbing, |
| (Sisani et al., 2019) | x | Mechanical sorting |
| (Behrooznia et al., 2020) | Selected as transport, freight, lorry 7.5–16 t. MSW is transported by special trucks to a compost plant located 10 km from the city | Sorting |
| (Francini et al., 2020) | Door-to-door collection: The transportation distances of the compost refining discards to the landfill and the liquid effluent to the wastewater treatment plant are presumed to be 100 km. | Shredder, electromagnet, rotating drum screen |
| (Lee et al., 2020) | For the FW and YW collection, single-unit refuse trucks (full capacity: 10 tons) fueled by diesel were used, and the average travel distance was 340 km/day/vehicle. For the collection, it was assumed that the trucks travelled at their maximum capacity for the entire distance, and the collection trucks ran five days per week. Transportation data were assumed as trailer trucks (full capacity: 20 tons for FW and YW and 32 tons for biosolids). The average transportation distance from the transfer stations and wastewater treatment facilities to landfilling and composting facilities was 93 km, and 45 km to HS-AcD (High-Solids Anaerobic co-digestion). | Shredder |
| (Mayer et al., 2020) | Municipal waste collection service by 21 metric ton lorry six t.km | Drying |
| (Morero et al., 2020) | x | X |
| (Chen et al., 2021) | x | Sorting, crushing, pulping, moisture-heat, and three-phase separation |
| (Sardarmehni and Levis, 2021) | The MSW collection model is based on (M.K. Jaunich, J.W. Levis, M.A. Barlaz, J.F. DeCarolis Lifecycle process model for municipal solid waste collection J. Environ. Eng., 142 (2016), Article 04016037, 10.1061/(ASCE)EE.1943-7870.0001065) which reported consumption of 10.1 litres diesel per Mg of residual waste collected based on the average data from two cities in the U.S. | Grinding, Separation |
| (Stobernack et al., 2021) | The transportation distance between the point of waste collection and the lignite power plant was varied between 0 (shortest distance), 222 km (average weighted distance), and 612 km (longest transportation distance between any point in Germany to the closest lignite power plant). The waste collection route was set to 5 km, and the distance to the first treatment plant was 40 km. | OFMSW stored immediately. The following step removes impurities and structural material and mixes the substrate with water. |
| (Tominac et al., 2021) | The transportation network includes the city transport stations. | Screening, mixing, dewatering |
| (Tyagi et al., 2021) | The system boundary does not consider the transportation of the MSW from different collection centres to the MBT plant. The transport of the waste to the landfill sites has been included in the system boundary. | Sorting, screening, shredder |
| (Wang et al., 2021) | The distance between the slaughterhouse and the AD plant/composting plant was assumed to be approximately 156 km. | Grinding, storage, equalisation |
| (Lara-Topete et al., 2022) | Vehicles, including their trajectories, from the collection point to the transfer station and then to the treatment facilities. Collection trucks, which collect MSW from the generation point and transport it to a transfer station with an average capacity of 8.2 tons. heavy-duty trucks, with an average capacity of 16 tons collect the MSW from the transfer stations and transport it to the landfill. Scenario A considers that all the waste is disposed of in a sanitary landfill at an average distance of 21.5 km away, while scenarios B and C consider disposal in a new site, which is an average distance of 95.5 km away | Manual separation, rotary drum, optic separators, magnetic separators, Foucault separators, RDF (refuse-derived fuel) production |
| (Le Pera et al., 2022) | Starting from the collection of food waste to the agricultural use of compost | Drum, mixing |
| (Mancini et al., 2022) | 50 km for the transport of OFMSW to the plant. | I* |
| (Weligama Thuppahige and Babel, 2022) | x | x |
| (Xiao et al., 2022) | In the six scenarios, it was assumed that FW was collected from households using a diesel truck with a capacity of 24.7 tons. The average distance from households to each treatment facility was approximately 20 km. | x |
| (Bottausci et al., 2023) | Lorry 16–32 t, 95,923.2 tkm. | Shredding, Mechanical shovels |
| (Castellani et al., 2023) | Lifespan: 5000,000 km. Maintenance every: 10,000 km. | A bag opener, a magnetic separator, a disc sieve and a shredder |
| (Gadaleta et al., 2023) | x | I* |
| (Guillaume et al., 2023) | Municipal services provided the transport distances, vehicle types, and fuel consumption. | x |
| (Le Pera et al., 2023) | SC-FW produced by the municipalities was transported to the industrial plant. | Bag opener and a solid waste separator, shredder |
| (Lewerenz et al., 2023) | Transport distances are estimated at 15 km (for the waste collection service (garbage trucks)) and 17 km from the collection point to the disposal point. | x |
| (Nyitrai et al., 2023) | x | x |
| (Rotthong et al., 2023) | The distance was measured from the first collection point to the transfer station. The collection of MSW in the city was around 3000 tons/day, and the collection distance was 17.1 km. The collection of MSW at the township municipality was around 6000 tons/day, and the collection distance was 17.5 km. The amount of MSW transport in the city was around 600 t/day. The average distance was measured from the transfer station to the treatment plants (18.7 km). | x |

Table S4: Detailing of data used in inventories for composting and AD process.

| Articles | Composting | Anaerobic digestion |
| --- | --- | --- |
| (Edwards et al., 2018) | I* | I* |
| (Hadzic et al., 2018) | X | Use of machinery, fossil energy consumption, electricity consumption, air emission, water emission, soil emission |
| (Cobo et al., 2019) | Use of machinery, fossil energy consumption, electricity consumption, air emission, windrow composting, tunnel composting | The use of machinery, fossil energy consumption, electricity consumption, air emission, leachates, and electricity generated in the biogas combustion process is 2.64 kwh·m^-3^ CH_4_ |
| (Francini et al., 2019) | Use of machinery, fossil energy consumption, electricity consumption, air emission, water emission, soil emission, mechanical sorting, screening, and Exhaust air is processed by biofiltration | Fossil energy consumption, electricity consumption, air emission, water emission, soil emission, leachates, s, chemicals and manufactured materials, energy production from biogas and hydrogen-rich gas, dewatering and centrifugation treatment of the AD residues, Polyelectrolite, NaOH (removal H2S by absorption) |
| (Mancini et al., 2019) | Use of machinery, fossil energy consumption, electricity consumption, air emission, null | Electricity consumption, air emission, electricity production |
| (Sisani et al., 2019) | Fossil energy consumption, air, water, soil, and chemicals emissions. I* not detailed | Fossil energy consumption, air, water, soil, and chemicals emissions. I* not detailed |
| (Behrooznia et al., 2020) | Use of machinery, fossil energy consumption, electricity consumption, air emission, water emission, soil emission, windrow | Fossil energy consumption, electricity consumption, water consumption, chemical inputs, internal combustion engine (CHP) |
| (Francini et al., 2020) | Fossil energy consumption, electricity consumption, air emission, aerated (turned) windrow | Fossil energy consumption, electricity consumption, air emission, leachates |
| (Lee et al., 2020) | Use of machinery, fossil energy consumption, electricity consumption, water consumption, forced aeration, air emission, water emission, leachates, Windrows | Use of machinery, fossil energy consumption, electricity consumption, air emission, leachates, CHP, Electricity production, and leachate were recirculated to the bioreactor, |
| (Mayer et al., 2020) | Use of machinery, fossil energy consumption, electricity consumption, water consumption, air emission, water emission, soil emission | Use of machinery, fossil energy consumption, electricity consumption, water consumption, air emission, water emission, soil emission |
| (Morero et al., 2020) | Use of machinery, fossil energy consumption, electricity consumption, air emission, windrow | Electricity consumption, water consumption, air emission, CHP (combined heat and power), electricity production |
| (Chen et al., 2021) | Electricity consumption, I* not detailed | Electricity consumption, I* not detailed |
| (Sardarmehni and Levis, 2021) | Use of machinery, fossil energy consumption, electricity consumption, air emission, water emission, soil emission, leachates | Use of machinery, fossil energy consumption, electricity consumption, air emission, water emission, soil emission, leachates |
| (Stobernack et al., 2021) | Air emissions tunnel composting plant. The I* is not detailed. | Air emissions dry fermentation. Produced biogas was combusted in a CHP plant. |
| (Tominac et al., 2021) | Fossil energy consumption, electricity consumption, windrow composting | Fossil energy consumption, electricity consumption, water consumption, and Biogas produced from the system is converted into electricity for the grid. |
| (Tyagi et al., 2021) | Use of machinery, fossil energy consumption, in-vessel composting drum and after stored in windrows. After 14 days, for maturation purposes, it is screened and bagged for further sale. | Machinery, fossil energy consumption, air emission, leachates, and bio-scrubber are used to remove hydrogen sulfide and then through a chiller unit to remove moisture. Generate electricity through biogas engines (2 units x 170 kW, total 340 kW capacity). Waste heat from the gas engines is used to heat the digesters. The treated effluent from ETP is used for gardening, cleaning requirements during operation and maintenance of the facility, flushing at the premises of the MBT plant |
| (Wang et al., 2021) | Use of machinery, fossil energy consumption, electricity consumption, water consumption, air emissions storage | Use of machinery, fossil energy consumption, electricity consumption, air emissions biogas storage, biogas cleaning, and combined heat and power (CHP) |
| (Lara-Topete et al., 2022) | Fossil energy consumption, electricity consumption, air emission | X |
| (Le Pera et al., 2022) | Fossil energy consumption, electricity consumption, air emission, leachates, s, Also electricity supplied by the photovoltaic system located on the roof of the buildings. The exhaust air produced during the ACT and maturation phases is conveyed and sent first to a sulphuric acid scrubber, to recover ammonium sulfate, and then to a vegetal biofilter before being released into the atmosphere. Liquid waste generated by the pretreatment section and the composting process is sent to the facility's wastewater treatment system, which generates clean water reused in the plant processes or discharged into the public sewer. | Fossil energy consumption, electricity consumption, air emissions Also electricity supplied by the photovoltaic system located on the roof of the buildings. Biogas is cleaned using a chemical scrubber using FeCl_3_ and NaOH, passed through an activated carbon system and upgraded to biomethane by a membrane separation unit. The biomethane obtained is fed into the natural gas network for use in the transport sector |
| (Mancini et al., 2022) | I* not available | I* not available |
| (Weligama Thuppahige and Babel, 2022) | Use of machinery, fossil energy consumption, electricity consumption, water consumption, air emission, water emission, soil emission, leachates, windrow composting | Electricity consumption, water consumption, air emission, water emission, soil emission, electricity production |
| (Xiao et al., 2022) | Electricity consumption, air emission, water emission, open windows | Fossil energy consumption, electricity consumption, water consumption, air emission, water emission |
| (Bottausci et al., 2023) | Use of machinery, fossil energy consumption, electricity consumption, air emission, leachates, Leachate to wastewater treatment | X |
| (Castellani et al., 2023) | Use of machinery, fossil energy consumption, electricity consumption, water consumption, air emission, water emission, turning piles | Fossil energy consumption, electricity consumption, water consumption, air emission, water emission |
| (Gadaleta et al., 2023) | Fossil energy consumption, electricity consumption, water consumption, air emission, leachates, null | Fossil energy consumption, electricity consumption, water consumption, air emission, leachates |
| (Guillaume et al., 2023) | Fossil energy consumption, electricity consumption, water consumption, air emission, water emissions windrow composting | Electricity consumption, air emission, water emissions fecl3, combined heat and power (CHP), electricity, heat and biogas are produced |
| (Le Pera et al., 2023) | Electricity consumption, water consumption, Water for scrubber and biofilter (m^3^), gaseous emissions, biofilter (mg/Nm^3^), compost | Electricity consumption, water consumption, Water for scrubber and biofilter (m^3^), gaseous emissions, biofilter (mg/Nm^3^), biomethane |
| (Lewerenz et al., 2023) | Use of machinery, fossil energy consumption, electricity consumption, forced aeration, air emission, water emission, composting open (“C, open”), closed (“C, closed”), partly closed (“C, partly closed”) and covered with a semipermeable membrane (“C, a membrane”) | Use of machinery, fossil energy consumption, electricity consumption, water consumption, air emission, water emission |
| (Nyitrai et al., 2023) | Use of machinery, fossil energy consumption, electricity consumption, water consumption, air emission | Electricity consumption, water consumption, air emission, NaOH, CHP, electricity production |
| (Rotthong et al., 2023) | Use of machinery, fossil energy consumption, water consumption, air emission, water emission, leachates, Molasses, Sawdust | Use of machinery, fossil energy consumption, electricity consumption, water consumption, air emission, water emission, Sodium hydroxide (NaOH) and calcium hydroxide (Ca (OH)_2_) |
| X: not included.  I*: Inventory is not detailed or available. | | |


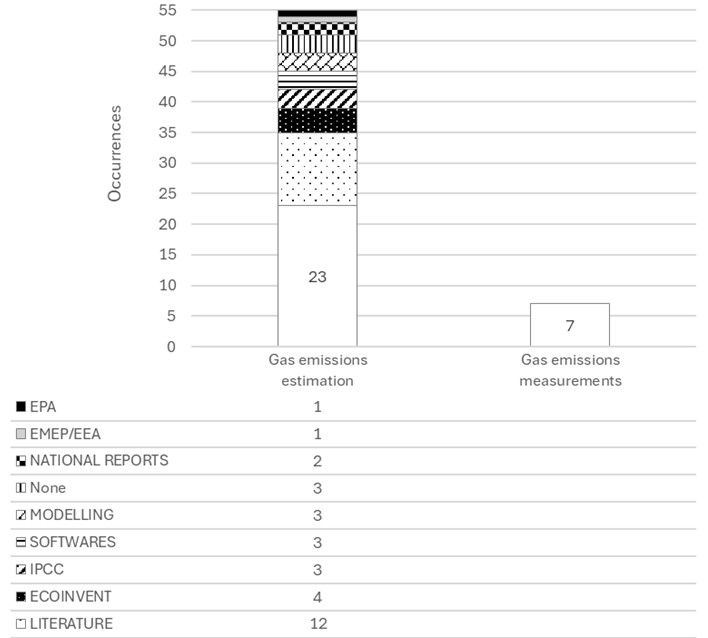


Figure S7: Number of studies that estimated gas emissions through reference sources and those that measured emissions.

EPA: Environmental Protection Agency; EMEP/EEA: European Environment Agency.
